# Genome Sequence of *Streptomyces albulus* PD-1, a Productive Strain for Epsilon-Poly-l-Lysine and Poly-l-Diaminopropionic Acid

**DOI:** 10.1128/genomeA.00297-14

**Published:** 2014-04-17

**Authors:** Zhaoxian Xu, Jun Xia, Xiaohai Feng, Sha Li, Hong Xu, Fangfang Bo, Zhuzhen Sun

**Affiliations:** a State Key Laboratory of Materials-Oriented Chemical Engineering, Nanjing, China; b College of Food Science and Light Industry, Nanjing University of Technology, Nanjing, China

## Abstract

*Streptomyces albulus* PD-1, a productive strain for epsilon-poly-l-lysine and poly-l-diaminopropionic acid, was isolated from soils. We present the genome sequence of *S. albulus* PD-1, which may provide abundant information regarding the production of epsilon-poly-l-lysine and poly-l-diaminopropionic acid.

## GENOME ANNOUNCEMENT

Epsilon-poly-l-lysine (ε-PL) was discovered by Shima and Sakai when they screened for a Dragendorff-positive substance from microbial origins (1). Composed of l-lysine residues linked by bonds between the α-carboxyl and ε-amino groups, ε-PL can be biosynthesized by *Streptomycetaceae* and ergot fungi (2, 3). The most prominent feature of ε-PL is its antimicrobial activity against a spectrum of microorganisms, including bacteria, fungi, and particular viruses (4). Therefore, ε-PL is used as a food preservative in many countries, such as Japan, the United States, and Korea. Moreover, the biodegradability, water solubility, and cationic structure of ε-PL make it a functional material with a bright development prospect in fields like medicine and electronics (5). *Streptomyces albulus* is the most common ε-PL-producing species. We previously isolated a highly efficient strain, *S. albulus* PD-1, from soils and deposited it in the China Center for Type Culture Collection (accession no. M2011043). Unlike other ε-PL producers, *S. albulus* PD-1 is capable of producing an additional amino acid oligomer with antimicrobial activity, poly-l-diaminopropionic acid, during fermentation (6). It is therefore interesting to explore the genetic composition of *S. albulus* PD-1 to help account for its physiological superiority in terms of ε-PL production and the mechanism leading to by-product generation.

In this study, we present the draft genome of *S. albulus* PD-1. The genome was extracted using a G^+^ bacterial genomic DNA kit and broken down via ultrasonic fragmentation. We obtained fragments of 300 bp, constructed the genomic library using a TruSeq DNA sample prep kit-Set A (Illumina), amplified the genome by using a TruSeq PE cluster kit (Illumina), sequenced the sample using the Illumina instrument (491-fold coverage), and assembled the sample using Velvet 1.2.10 (7, 8). This assembly produced 244 contigs, with an *N*_50_ of 66,972 bp. As submitted to GenBank, the draft genome sequence of strain *S. albulus* PD-1 contains 9,427,044 bases with a mean G+C content of 72.3%. The genome sequence was annotated using the NCBI Prokaryotic Genomes Automatic Annotation Pipeline (9), resulting in 8,090 genes, 3 rRNAs, and 67 tRNAs. The contigs were searched against the Kyoto Encyclopedia of Genes and Genomes (KEGG) and Clusters of Orthologous Groups databases to analyze gene functions and metabolic pathways (10).

Based on KEGG pathway analysis, most of the genes that encode proteins for metabolic, genetic, and environmental information processing were successfully annotated. Similar to those of most other *Streptomyces* species, the central carbon metabolism of *S. albulus* PD-1 includes glycolysis, the tricarboxylic acid cycle, the pentose phosphate pathway, and the anaplerotic metabolic pathway (11). Like that in *S. albulus* strain NBRC14147 (12), l-lysine in *S. albulus* PD-1 is biosynthesized through the amino acid biosynthetic pathway from l-aspartic acid and is terminated by ε-PL synthetase. Investigating the genome of *S. albulus* PD-1 may yield further insights into its considerable metabolic potential and may provide more strategies to control the fermentation of ε-PL.

### Nucleotide sequence accession numbers.

This whole-genome shotgun project has been deposited at DDBJ/EMBL/GenBank under the accession no. AXDB00000000. The version described in this paper is version AXDB02000000.
